# A “Lymphocyte MicroRNA Signature” as Predictive Biomarker of Immunotherapy Response and Plasma PD-1/PD-L1 Expression Levels in Patients with Metastatic Renal Cell Carcinoma: Pointing towards Epigenetic Reprogramming

**DOI:** 10.3390/cancers12113396

**Published:** 2020-11-16

**Authors:** Lorena Incorvaia, Daniele Fanale, Giuseppe Badalamenti, Chiara Brando, Marco Bono, Ida De Luca, Laura Algeri, Annalisa Bonasera, Lidia Rita Corsini, Salvatore Scurria, Juan Lucio Iovanna, Antonio Russo, Viviana Bazan

**Affiliations:** 1 Department of Biomedicine, Neuroscience and Advanced Diagnostics (Bi.N.D.), Section of Medical Oncology, University of Palermo, 90127 Palermo, Italy; lorena.incorvaia@unipa.it (L.I.); viviana.bazan@usa.net (V.B.); 2 Department of Surgical, Oncological and Oral Sciences, Section of Medical Oncology, University of Palermo, 90127 Palermo, Italy; fandan@libero.it (D.F.); giuseppe.badalamenti@unipa.it (G.B.); chiabra92@libero.it (C.B.); marco.bono@unipa.it (M.B.); ida.deluca@unipa.it (I.D.L.); laura.algeri@community.unipa.it (L.A.); annalisa.bonasera@unipa.it (A.B.); lidiarita.corsini@community.unipa.it (L.R.C.); 3 Department of Surgical, Oncological and Oral Sciences, Section of Urology, University of Palermo, 90127 Palermo, Italy; salvatore.scurria@hsrgiglio.it; 4 Team Pancreatic Cancer, Centre de Recherche en Cancérologie de Marseille (CRCM), INSERM U1068, CNRS UMR 7258, Aix-Marseille Université and Institut Paoli-Calmettes, Parc Scientifique et Technologique de Luminy, 13288 Marseille, France; juan.iovanna@inserm.fr

**Keywords:** microRNA, miRNA, PD-1, PD-L1, predictive biomarkers, renal cell carcinoma, soluble immune checkpoints

## Abstract

**Simple Summary:**

MicroRNAs are small molecules of non-coding RNAs which regulate gene expression at the post-transcriptional level. Normal miRNA expression and function can be deregulated in cancer. The comprehensive molecular characterization of Renal Cell Carcinoma shows several genes silenced and signaling pathways deregulated by epigenetic modifications, such as the abnormal expression of miRNAs. They can be secreted from malignant cells in whole-blood, plasma, serum, and urine samples, making miRNAs potential non-invasive tumor biomarkers. However, if a single miRNA can show low discriminatory power, the combination of miRNAs in a “miRNA signature”, identified in the peripheral lymphocytes of patients, could function better with much higher probability to predict the response to immunotherapy and to discriminate responders from non-responders patients already at therapy baseline.

**Abstract:**

Introduction of checkpoint inhibitors resulted in durable responses and improvements in overall survival in advanced RCC patients, but the treatment efficacy is widely variable, and a considerable number of patients are resistant to PD-1/PD-L1 inhibition. This variability of clinical response makes necessary the discovery of predictive biomarkers for patient selection. Previous findings showed that the epigenetic modifications, including an extensive microRNA-mediated regulation of tumor suppressor genes, are key features of RCC. Based on this biological background, we hypothesized that a miRNA expression profile directly identified in the peripheral lymphocytes of the patients before and after the nivolumab administration could represent a step toward a real-time monitoring of the dynamic changes during cancer evolution and treatment. Interestingly, we found a specific subset of miRNAs, called “lymphocyte miRNA signature”, specifically induced in long-responder patients (CR, PR, or SD to nivolumab >18 months). Focusing on the clinical translational potential of miRNAs in controlling the expression of immune checkpoints, we identified the association between the plasma levels of soluble PD-1/PD-L1 and expression of some lymphocyte miRNAs. These findings could help the development of novel dynamic predictive biomarkers urgently needed to predict the potential response to immunotherapy and to guide clinical decision-making in RCC patients.

## 1. Introduction

Renal cell carcinoma (RCC) represents a heterogeneous group of cancers where our understanding of genetic and molecular drivers, clinical behavior, and responses to therapy have evolved over the past years, changing the clinical landscape and the natural history of the disease [1].

The introduction of checkpoint inhibitors resulted in durable responses and improvements in overall survival (OS) in advanced RCC patients [2,3,4,5], but the treatment efficacy is widely variable, and a considerable number of patients are resistant to PD-1/PD-L1 inhibition. This variability of clinical response to immunotherapy makes necessary the discovery of predictive biomarkers for patient selection. PD-L1 status on tumor tissue has been shown to be an imperfect predictive biomarker and, despite the intensive effort, reproducible and dynamic biomarkers to select those patients who are most likely to have a good immunotherapy response have not yet been developed for therapeutic decisions [6].

Since previous findings showed that the epigenetic modifications are a key feature of RCC [7], investigating the epigenetic reprogramming, such as the extensive microRNA (miRNA)-mediated regulation of tumor suppressor genes, could represent a step toward a real-time monitoring of the dynamic changes during cancer evolution and treatment, as well as the effects of the complex interplay between tumor cells and the immune system [8].

miRNAs are small molecules (19–22 nucleotides) of non-coding RNA that regulate gene expression at the post-transcriptional level through binding to complementary nucleotides in the 3’ untranslated region (UTR) of messenger RNA (mRNA) target [9,10]. miRNAs silence the gene expression by repressing translation and accelerating target mRNA degradation. Normal miRNA expression and function can be deregulated in cancer [11,12]. Their critical roles in cancer find evidence in a complex map of interactions underlying the relationship between miRNA regulation and the hallmarks of cancer [13]. A single miRNA can target multiple mRNAs and one mRNA can also be targeted by multiple miRNAs [14]. As a consequence, the aberrant expression of miRNAs can affect a multitude of transcripts and different cancer-related signaling pathways [15,16,17].

Moreover, analysis of gene expression signatures in clear cell RCC (ccRCC) reveals a high expression of several genes involved in immune checkpoint pathways, including PD-1 and PD-L1 genes, known as key targets for immunotherapy [18]. Emerging evidence reports that the PD-L1 and PD-1 levels are strongly controlled by the miRNA network, which seems, therefore, to have a profound regulatory effect on the expression of specific immune checkpoint-related genes through a complex regulatory mechanism [18].

Studies on miRNAs from tumor patients showed a wide stability in various tissues and body fluids because they can be secreted from malignant cells in whole-blood samples, plasma, serum, and urine, making miRNAs potential non-invasive diagnostic, prognostic, and predictive tumor biomarkers [19]. Finally, synergy of two or more miRNAs has been shown to possess great clinical value, supporting the development of combinatorial miRNA signatures (multiplex miRNA panels) as clinical tools.

Despite these appealing findings, no epigenetic biomarkers are currently used in the clinic. With this goal, we performed a prospective study in renal cancer patients in order to analyze the peripheral lymphocyte miRNA expression profile in long-responder patients to nivolumab treatment, with the aim to identify a “lymphocyte miRNA signature” predictive of an immunotherapy response. Furthermore, focusing on the clinical translational potential of miRNAs in controlling the expression of immune checkpoints, we investigated the association between the plasma levels of soluble PD-1/PD-L1 and expression of some lymphocyte miRNAs.

## 2. Results

### 2.1. Patients Characteristics

Twenty-three (23) metastatic ccRCC patients were included in the study. All the patients were treated with second-line nivolumab. The clinical and pathological characteristics of the study population are summarized in Table 1.

### 2.2. Expression Profile of Lymphocyte miRNAs as Predictive Biomarkers of Anti-PD-1 Treatment Outcome in Metastatic ccRCC Patients

A large-scale analysis of 377 miRNAs on peripheral blood samples from the 23 mccRCC patients was performed in order to investigate the effect of nivolumab treatment on the lymphocyte miRNA expression profile.

The lymphocyte miRNA expression profile, before nivolumab treatment (T0) and after a 4-weeks period (T1) with nivolumab treatment (2 cycles of nivolumab administration), was analyzed.

**Figure 1:**
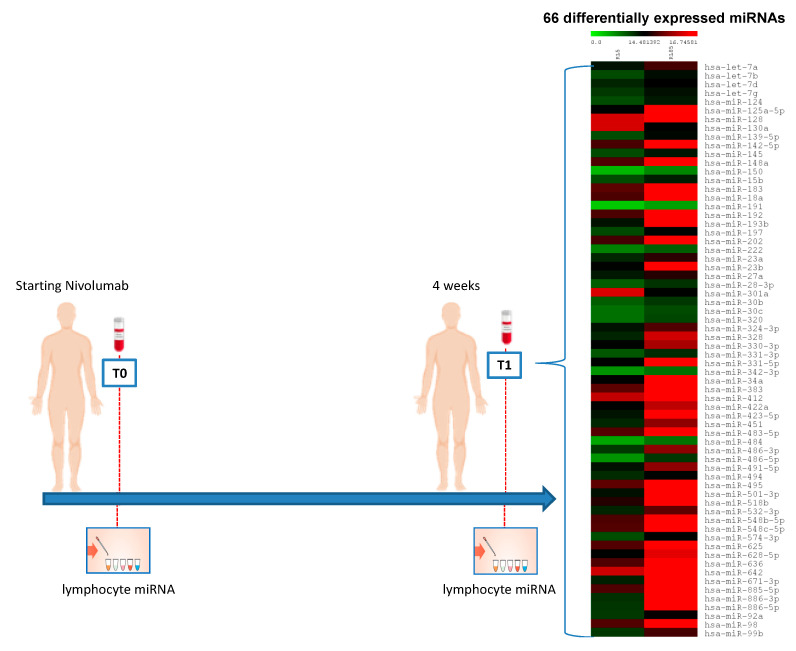
Microarray analysis of 377 miRNAs from 23 mRCC patients, analyzed before nivolumab treatment (T0) and after a 4-weeks period (T1) with nivolumab treatment (2 cycles of nivolumab administration), showing 66 differentially expressed miRNAs in peripheral lymphocytes at T1 vs. T0. Sixty-four miRNAs are downregulated and 2 are upregulated. The heat map of differentially expressed miRNAs was generated from microarray data reflecting mean expression values in 23 clear cell RCC (mccRCC) patients before treatment with nivolumab (T0) and 4 weeks after treatment (T1). Only upregulated miRNAs with fold change >2 and downregulated miRNAs with fold change < 0.3 were considered (*p* < 0.05). Each row represents the mean expression levels for a single miRNA tested at baseline and after treatment. Each column shows the mean expression levels for all miRNAs tested for a single patient group. The absolute expression value of each miRNA is derived from the mean Ct value calculated for each patient group. The color scale bar on the top represents signal intensity variations ranging from green (low mean Ct value and highly expressed miRNAs) to red (high mean Ct value and poorly expressed or unexpressed miRNAs). Black boxes indicate intermediate expression values. Data were obtained by TaqMan^®^ Low Density Array A Human MicroRNA using RNU48 as an endogenous control.

Among all 377 analyzed miRNAs, microarray analysis showed 66 differentially expressed miRNAs in peripheral lymphocytes between T0 and T1. Sixty-four miRNAs were upregulated, and only 2 miRNAs were downregulated at T1 versus T0. The 66 differentially expressed miRNAs are graphically represented in Figure 1.

To investigate the predictive role of lymphocyte miRNA expression profile in the immunotherapy response, we divided the patients based on the progression-free survival (PFS) to nivolumab treatment and best overall response by RECIST (complete response, CR; partial response, PR; stable disease, SD; progression disease, PD). Two of 23 patients showed a PFS < 6 months (Group A), 11/23 patients a PFS between 6–18 months (Group B), and 9/23 patients a PFS > 18 months (Group C). We compared the lymphocyte miRNA expression profile between three groups. In order to highlight the significantly expressed miRNAs, we established a cutoff of fold change (FC) > 2 for upregulated miRNAs and FC < 0.3 for downregulated miRNAs (*p* < 0.05).

After 4 weeks of nivolumab treatment (T1), the patients with SD, PR, or CR as the best response (Groups 1 and 2) showed 28 lymphocyte miRNAs specifically induced by nivolumab treatment (Table 2).

**Figure 2:**
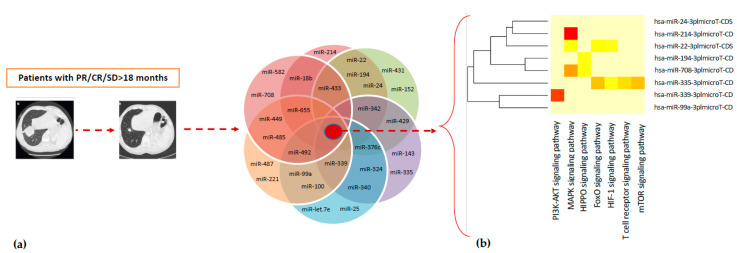
(**a**) Twenty-eight miRNAs were specifically induced by Nivolumab treatment in mRCC patients with PR/CR/SD as the best response; (**b**) miRNAs versus pathways heat map (clustering based on significance levels). The dendrograms placed on both axes depict hierarchical clustering results for miRNAs and pathways, respectively. On the miRNA axis, we can identify clustered miRNAs by exhibiting similar pathway targeting patterns. Eight miRNAs were specifically induced by Nivolumab treatment in long-responders (>12 months) mRCC patients. Hierarchical clustering was realized using DIANA-miRPath v3.0.

These 28 miRNAs were silenced at T0. Analysis through DIANA-mirPath v.3.0 tool showed that 21 out of 28 induced-therapy miRNAs were related to clear cell renal cancer signaling pathways (Figure 2, Table S1)

Subsequently, a statistical analysis, performed by assessing only the intersection of targeted genes (hypothetical genes targeted by all selected miRNAs), revealed the involvement of 26 out of the 28 miRNAs and 183 miRNA-targeted genes involved in the PI3K-Akt signaling pathway—one of the most significant pathways in cancer biology (Table 3).

In addition, a further investigation was performed on the miRNA expression profile data in order to identify other molecular pathways of particular interest. In fact, other miRNA-related pathways, such as MAPK signaling (130 genes, 24 miRNAs), T-cell receptor (55 genes, 24 miRNAs), Hippo signaling (87 genes, 22 miRNAs), FOXO signaling (75 genes, 22 miRNAs), HIF-1 signaling pathway (56 genes, 22 miRNAs), mTOR signaling (35 genes, 20 miRNAs), were identified (Table 3). Our analysis showed that miRNAs are potential molecular mediators through which nivolumab may exert its anticancer activity.

**Figure 3:**
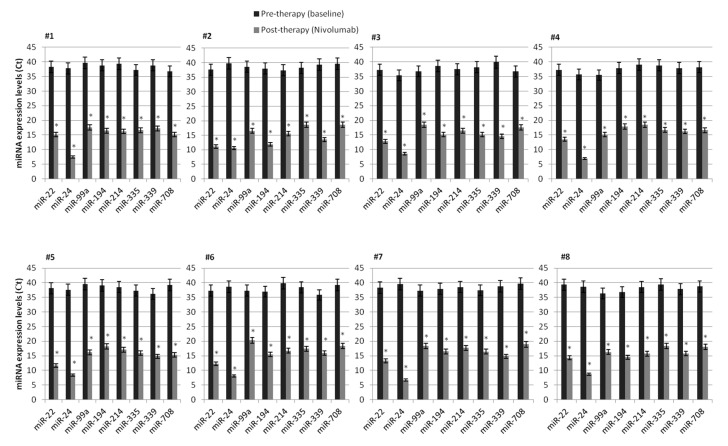
Validation of microarray expression data of 8 specific miRNA in a cohort of 8 mccRCC patients by quantitative Real-Time PCR analysis. The expression of each miRNA was analyzed before nivolumab treatment (T0) and 4 weeks after treatment (T1). Data are presented as Ct values ± SDs. Undetermined values of Ct were estimated between 35 and 40 Ct (the last cycle of the reactions), whereas a cut off <35 Ct was used to select reliably quantifiable miRNAs. RNU48 was used as an endogenous control. * *p* value < 0.0001.

In order to investigate the predictive role of deregulated lymphocyte miRNAs, we evaluated the expression of some specific miRNAs exclusively induced after nivolumab treatment in long-responder patients, recognized as patients with CR/PR or SD to nivolumab >18 months. Interestingly, in this subgroup of patients, a subset of 8 specific miRNAs, such as miR-22, miR-24, miR-99a, miR-194, miR-214, miR-335, miR-339, miR-708, strongly induced by nivolumab treatment, was identified. This “lymphocyte signature” of 8 miRNAs has been found to be specifically and highly induced by nivolumab treatment and present only in peripheral lymphocytes from long-responder mRCC patients (>18 months). In fact, before nivolumab treatment (T0), these miRNAs are resulted to be silenced, but their expression was surprisingly restored after 4 weeks of treatment (T1) (Table 4), suggesting, therefore, an association with the duration of response to anti-PD-1 therapy. These results were confirmed through quantitative Real-Time PCR analysis in an independent validation cohort of 8 mccRCC patients (Figure 3). An enrichment analysis carried out using online tools available from The Database for Annotation, Visualization and Integrated Discovery (DAVID) allowed us to identify 41 KEGG pathways modulated by the “lymphocyte signature” of 8 miRNAs, including several signaling pathways related to renal cell carcinoma (Figure S1).

The table represents a list of 8 out of 28 miRNAs specifically induced by Nivolumab in mRCC patients. The first column reports the specifically induced miRNAs, the middle column shows mean Ct value and the last column reports gene targets related to miRNAs. Data were obtained by TaqMan^®^ Low Density Array A Human MicroRNA using RNU48 as an endogenous control.

### 2.3. Association Between Lymphocyte miRNA Expression and Plasma Levels of Soluble PD-1/PD-L1 in Long-Responders Patients

**Figure 4:**
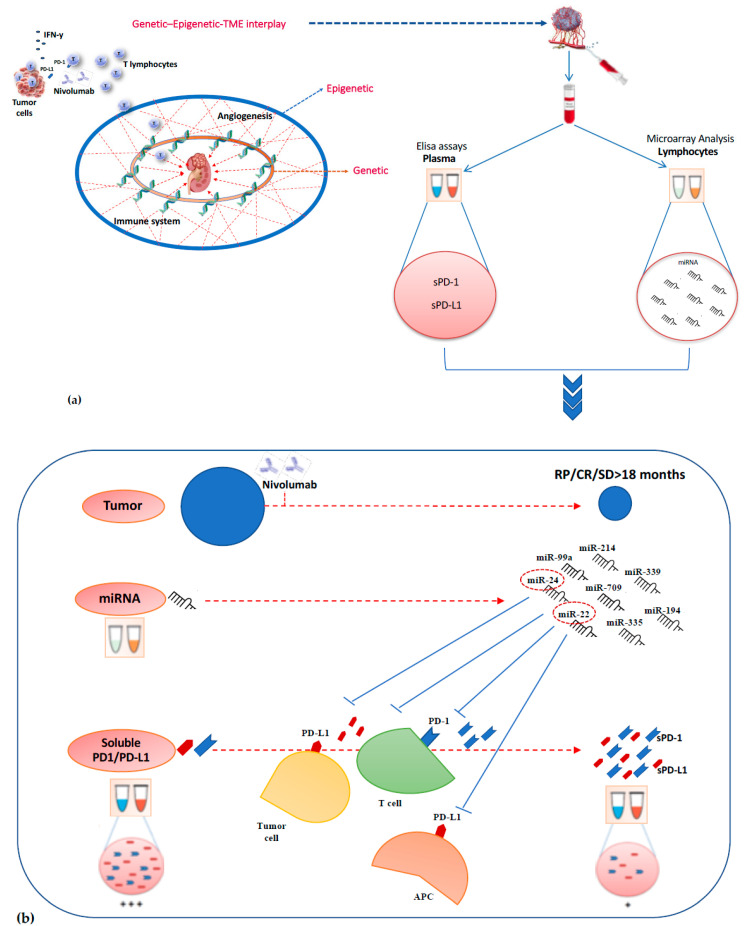
(**a**) We investigated the association between plasma levels of soluble PD-1/PD-L1 and lymphocyte miRNA expression profile in long-responder mccRCC patients. (**b**) miR-22 and miR-24 levels were inversely associated with plasma PD-1 and PD-L1 levels in long-responder patients to nivolumab treatment. At baseline, high sPD-1/sPD-L1 levels were observed, whereas the expression of lymphocyte miR-22/miR-24 was silenced. After 4 weeks from starting nivolumab, sPD-1/sPD-L1 levels were strongly reduced and the expression of miR-22/miR-24 was restored only in patients with PR/CR/SD to nivolumab >18 months.

To investigate the translational potential of miRNAs in regulating the immune checkpoint expression, the association between plasma levels of soluble PD-1/PD-L1 (sPD-1 and sPD-L1) and lymphocyte miRNA expression profile was studied in 9 long-responder patients to nivolumab treatment (Figure 4). In this patient cohort, we showed that miR-22 and miR-24 levels were inversely correlated, in a statistically significant way, with plasma PD-1 levels. At baseline (T0), high sPD-1 levels were observed (median 13.15 ng/mL; range: 1.12–25.00), whereas the expression of lymphocyte miR-22/miR-24 was silenced (Ct > 40). Conversely, after 4 weeks from starting nivolumab (T1), sPD-1 levels were strongly reduced (median 1.25 ng/mL; range: 1.06–1.97) and the expression of miR-22/miR-24 was restored (mean Ct: 12.86 and 7.80, respectively) only in patients with PR/CR/SD to nivolumab >18 months (*p* = 0.007), suggesting that a miRNA network could inhibit sPD-1 expression mainly via miR-20 family.

**Figure 5:**
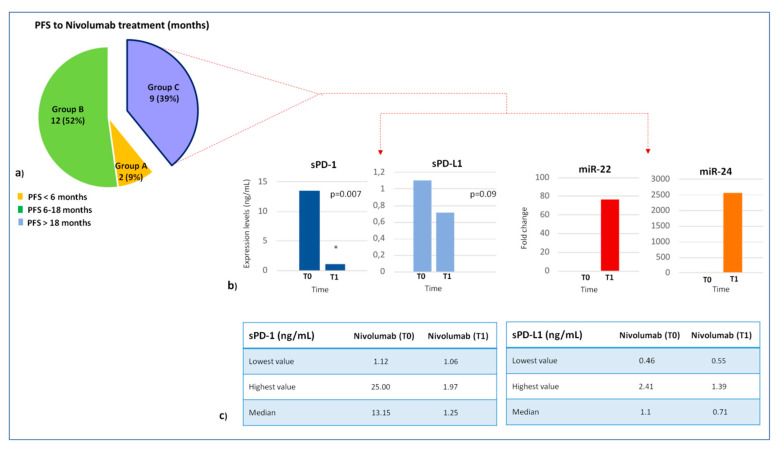
Correlation between expression of miRNAs 22/24 and plasma levels of soluble PD-1/PD-L1 in a group of 9 long-responder patients. (**a**) Pie chart representation of 23 patients receiving nivolumab treatment divided by PFS: 2 patients with PFS < 6 months (group A); 12 patients with PFS between 12−18 months (group B) and 9 patients with PFS > 18 months (group C). (**b**) Comparison between plasma levels of PD-1/PD-L1 and miRNA 22/24 expression before nivolumab treatment (T0) vs. 4 weeks later (T1). The histograms of PD-1 and PD-L1 correlate the expression level of immune checkpoints with time. The histograms of microRNA 22/24 correlates the value of fold change in relation to time. * *p* value < 0.01 (**c**) Tables represent the concentrations of immune checkpoints PD-1 and PD-L1 in 9 patients at T0 e T1.

In the same way, an inverse correlation between sPD-L1 and lymphocyte miR-22 and miR-24 was showed (T0, median value: 1.1 ng/mL; range 0.46–2.41; T1, median value: 0.71 ng/mL; range 0.55–1.39), but, probably, the small number of analyzed samples did not allow us to demonstrate a statistically significant difference between T0 and T1 (*p* = 0.09) (Figure 5).

## 3. Discussion

The treatment paradigm for mRCC continues to evolve in parallel to new knowledge on molecular biology and immunological background of renal tumors [20]. The immune checkpoint inhibitors are now routinely used in clinical practice, but recognizing which patient will benefit from therapy through a non-invasive biomarker still remains poorly achievable in daily clinical practice [21]. Epigenetic modifications are emerging as central features of renal cancers. The numerous genetic, genomic, and immunological alterations caused by the tumor cells can be in part reflected by altered gene expression profiles. According to the hypothesis of the importance of the epigenetic mechanisms in RCC, the comprehensive molecular characterization of RCC shows several genes silenced and signaling pathways deregulated by epigenetic modifications, such as the aberrant DNA methylation or abnormal expression of miRNA [7]. This event could represent a key mechanism for gene repression also in the absence of detectable mutations [14]. Furthermore, previous studies using the combination of histology plus genomics TCGA data revealed sporadic mutations of at least one out of nine genes associated with ccRCC in 81% of tumor tissue. It has been observed that, with the exception of *VHL*, mutated genes, such as *PBRM1*, *SETD2,* and *BAP1*, are mostly involved in histone modification and chromatin remodeling. These chromatin modifier genes located at chromosome 3p, when mutated, may lead to an altered epigenetic control of gene expression, contributing to the development and progression of RCC [22].

Based on this biological background, we hypothesized that a miRNA expression profile directly identified in the peripheral lymphocytes of the patients before and after the immunotherapy administration could be useful to evaluate the dynamic molecular changes underlying the nivolumab therapy and predict the treatment response.

Through microarray analysis, our investigation showed several differentially expressed miRNAs in peripheral lymphocytes of long-responder patients, most of which are implicated in key RCC signaling pathways involving VHL-HIF, PI3K/Akt, MAPK cascade, mTOR, FOXO, and T-cell receptor.

Interestingly, we found a specific subset of miRNAs (miR-22, miR-24, miR-99a, miR-194, miR-214, miR-335, miR-339, miR-708), which we called “lymphocyte miRNA signature”, specifically induced in long-responder patients recognized as the patients with CR, PR, or SD to nivolumab >18 months. These miRNAs were silenced at baseline in mccRCC patients, but exceptionally expressed in long-responders, already after 4 weeks of treatment with nivolumab. This mechanism of miRNA restoration in responder patients could be useful for identifying potential predictive biomarkers of therapy efficacy and nivolumab response.

Our results have been shown to be perfectly congruent with the recent findings reported in the literature concerning changes in expression of 8 therapy-induced miRNAs involved in cell proliferation, cell cycle regulation, apoptosis, migration, and invasion of RCC. In fact, our analysis revealed a specific nivolumab-mediated induction of the miR-22 expression, which is coherent with recent studies showing that this miRNA is downregulated both in serum and tissues of ccRCC patients and, therefore, it can act as tumor suppressor, since its loss of expression could contribute to the RCC development via the PTEN-mediated induction of cell proliferation, migration, and invasion [23]. This data suggest that the miR-22/PTEN or miR-22/SIRT1 axes could be considered new potential therapeutic targets useful for the development of effective therapeutic strategies against RCC [24,25]. Instead, discordant results were reported in a recent work published by Gong and collaborators [26] showing that the miR-22 overexpression promotes the cell proliferation and invasion of primary ccRCC cells in vitro. Unlike our analysis which showed a high and specific induction of miR-24 after nivolumab treatment, few and conflicting data were reported for this miRNA in RCC [27,28].

A potential tumor-suppressive role mediated by mTOR pathway was attributed to miR-99a, since this miRNA has been shown to be strongly downregulated in renal cancer tissues and correlated with poor survival in RCC patients. Conversely, its overexpression has been observed to induce G1-phase cell cycle arrest and inhibit cell growth and tumorigenicity in RCC [29,30]. Additionally, Osako et al. [31] reported a marked reduction of the miR-99a expression levels via regulation of ribonucleotide reductase regulatory subunit-M2 (RRM2) in sunitinib-resistant ccRCC cell lines. This data further supports the reliability of our results, showing the specific nivolumab-mediated induction of the miR-99a expression. However, a most recent study by Oliveira and collaborators [32] described an increased expression of miR-99a and downregulation of its target gene mTOR in ccRCC tissue samples, hypothesizing a potential oncogenic role for this miRNA.

According to our results, miR-194 has been shown to be a favorable prognostic biomarker, since its expression was positively correlated with disease-free survival (DFS) and OS in 234 ccRCC patients [33]. In addition, other experimental evidence showed the role of this miRNA in the inhibition of the tumor progression and therapy resistance in RCC [34,35,36,37].

Also, miR-214 has been shown to inhibit proliferation of RCC cells through reduction in expression levels of Insulin-like Growth Factor-1 (IGF-1) Receptor and inhibition of downstream mTORC1 signaling, regardless of VHL status [38]. Conversely, low expression levels of miR-214 were associated with enhanced cell growth and reduced drug sensitivity in RCC cells [39].

Furthermore, Wang et al. [40] showed that the miR-335 upregulation suppresses the proliferation and invasion of ccRCC cells via direct repression of the *BCL-W* gene, whereas miR-335 downregulation is significantly associated with occurrence of lymph node metastasis and increased tumor size.

Further evidence supporting our results is the potential association observed by Liu and collaborators [41] between miR-339 downregulation and increased expression of tumor PD-L1, resulting in an attenuated antitumor immune response in RCC patients. Therefore, the induction of the miR-339 expression after nivolumab treatment is congruent with the longer PFS observed in our cohort of long-responder patients.

Lastly, miR-708 has been shown to induce apoptosis and inhibit cell growth, invasion, migration, and tumorigenicity in renal cancer cells and murine xenograft models of human RCC [42].

In general, this data supports the hypothesis that nivolumab-induced modifications of the miRNA expression profile contribute to the anticancer efficacy of this agent. However, conflicting data for some miRNAs may be the result of an unsuitable methodology or inadequate sampling of examined tumor specimens. Moreover, further investigations are needed to clarify the miRNA-mediated interplay between tumor cells and immune system in the patients with disease progression as the best response. These patients are under-represented in this study, probably due to a homogeneity in the clear cell histological subtype, with only one prior antiangiogenic therapy, differently from CheckMate 025 trial. As regards the clinical translational potential of miRNAs in modulating expression of immune checkpoints [43], we investigated the association between the lymphocyte miRNA signature identified in the long-responder RCC patients and the plasma levels of soluble PD-1 and PD-L1.

Recently, a molecular link between the evasion from the immune response by lung cancer cells and miRNA function has been identified. Chen et al. [44] demonstrated that miR-200 suppressed the epithelial to mesenchymal transition (EMT) process by targeting PD-L1 and thus delaying cancer progression in a mouse model. It has also been observed that miR-200 expression negatively correlates with PD-L1 expression, suggesting the potentiality of miRNA expression as a predictive biomarker for immunotherapy response [44]. Previous studies showed that baseline levels of soluble PD-1 and PD-L1 were associated with a longer PFS to nivolumab treatment in a cohort of metastatic ccRCC patients, and high sPD-1 were also associated with best overall response by RECIST and objective response of >20% [45,46,47]. In our patient cohort, the inverse correlation between miR-22 and miR-24 levels and plasma PD-1 levels in long-responder patients suggests that a miRNA network could inhibit the immune checkpoint expression, mainly via the miR-20 family.

Despite this potential, our study presents several limitations due to the highly variable effect of a single miRNA on the target transcripts and related to the relative promiscuity of their targets. A specific miRNA may have thousands of targets and, therefore, the repressive signal on their target genes can be small. This small size of the repressive effect, together with the difficulties of target prediction, make challenging, to date, the clinical application of single miRNAs in a clinical setting.

However, if a single miRNA can show low discriminatory power, the combination of miRNAs, such as those included in our “miRNA signature”, could function better with a much higher probability to predict a response to immunotherapy and to discriminate responder from non-responder patients already at therapy baseline. Further studies on larger sets of patients are necessary to confirm this preliminary data. Finally, because multiple combinations of VEGFR-TKI + anti-PD-1/PD-L1 mAbs are under development and some are already included in the current landscape of first line therapy, determining if our findings on the predictive role of “miRNA signature” will also be confirmed in this setting. This will be extremely relevant for treatment selection.

## 4. Patients and Methods

### 4.1. Study Population

This is a prospective study including all consecutive patients with histological diagnosis of metastatic clear cell renal cell carcinoma and radiological evidence of metastatic disease, candidate to the second-line treatment with nivolumab based on medical choice among the currently available drugs. A written informed consent was obtained from each recruited patient in the study (Protocol “G-Land 2017”) approved by the ethical committee (Comitato Etico Palermo 1; approval number: 0103-2017) of the University-affiliated Hospital AOUP ‘P. Giaccone’ of Palermo. All clinical information was anonymously recorded and coded.

Peripheral blood samples from ccRCC patients were prospectively obtained from April 2017 to February 2019. Blood samples were collected at baseline, before starting nivolumab treatment (T0), and after a 4-weeks period (T1, 2 cycles of nivolumab administration). In the blood samples miRNAs were extracted from peripheral lymphocytes. The expression profile of 377 lymphocyte miRNAs was analyzed, with a cut off of fold change >2 for upregulated and <0.3 for downregulated miRNAs. The clinical and pathological information collected included age, gender, histologic subtype, grading, clinical stage according to the TNM system of the American Joint Committee on Cancer (AJCC), Karnofsky performance status (PS), type of surgery, prognostic factors, site and number of metastases, tumor response [progression disease (PD), stable disease (SD), partial response (PR), complete response (CR)] assessed according to Response Evaluation Criteria In Solid Tumors (RECIST version 1.1.) and progression-free survival (PFS) to nivolumab treatment.

The correlation of expression profile of lymphocyte miRNAs with PFS was analyzed. Association between plasma levels of soluble PD-1/PD-L1 and expression of lymphocyte miRNAs was investigated in long-responder patients to nivolumab treatment.

### 4.2. Sample Collection and Lymphocytes Isolation

The peripheral blood samples from patients were processed within 2 h of collection, by centrifugation at 2.200 r.c.f. for 15 min at 4 °C in the presence of EDTA.

Blood samples from patients were obtained by venipuncture before starting chemotherapy and 4 weeks later for the isolation of lymphocytes.

After plasma isolation and separation, the 4 blood tubes, to which 3 mL of Lympholyte (Lympholyte-H cell separation media, CL5020, Cederlane) were added, were centrifuged at 800 r.c.f. for 20 min at 18 °C. After centrifugation, a white colored ring containing lymphocytes can be isolated in the interface. The isolated lymphocytes were aliquoted in cryotubes and stored at −80 °C until their use for subsequent analysis.

### 4.3. miRNA Expression Profile Analysis

Total cellular RNA and miRNAs have been isolated using the miRNeasy Mini Kit (Qiagen Inc, Valencia, CA, USA). The quality of the samples have been controlled through RNA 6000 Nano Assay (Agilent Technologies, Palo Alto, CA, USA) using 2100 Bioanalyzer (Agilent Technologies, Santa Clara, CA, USA) and quantified through the spectrophotometer NanoDrop ND-1000 (CELBIO). To study miRNA expression profile, we used TaqMan^®^ Low Density Array A Human MicroRNA v2.0 (Life Technologies, Carlsbad, CA, USA). Briefly, 600 ng of miRNA-enriched total RNA were reverse transcribed using Megaplex™ RT Primers Human Pool A (Life Technologies, Carlsbad, CA, USA) according to manufacturer’s instructions. Conditions for the reverse transcription reaction were the same previously used in other works [48,49,50].

Obtained cDNA was diluted, mixed with TaqMan Gene Expression Master Mix and loaded into each of the eight fill ports on the TaqMan^®^ Human MicroRNA Array A (Life Technologies, Carlsbad, CA, USA). The TaqMan Human MicroRNA Array is a 384-well microfluidics card containing 377 primer-probe sets for individual miRNAs as well as three carefully selected candidate endogenous small nucleolar RNAs control assay and one negative control assay. The array was centrifuged at 1200 rpm twice for 1 min each, then run on ABI-PRISM 7900 HT Sequence Detection System (Applied Biosystems, Foster city, CA, USA). The data were quantified using the SDS 2.4 software and normalized using the RNU48 as an endogenous control. The cycle threshold (Ct) value, which was calculated relatively to the endogenous control, was used for our analysis (ΔCt). The 2^−ΔΔCT^ (delta-delta-Ct algorithm) method was used to calculate the relative changes in miRNA expression. A miRNA was defined differentially expressed when estimated *p*-value was <0.05.

### 4.4. Quantitative Real-Time PCR

Ten nanograms of total RNA were reverse transcribed using Taqman microRNA Reverse Transcription Kit (Life Technologies, Carlsbad, CA, USA) according to manufacturer’s instructions, as previously described [51]. The obtained cDNA was amplified using the following Taqman MicroRNA assays: hsa-miR-22, hsa-miR-24, hsa-miR-99a, hsa-miR-194, hsa-miR-214, hsa-miR-335, hsa-miR-339, hsa-miR-708 (Life Technologies, Carlsbad, CA, USA).

To normalize quantitative Real-Time PCR reactions, parallel reactions were run on each sample for RNU48 snRNA. The reactions were performed in triplicate and changes in the target miRNA content relative to RNU48 were determined using the comparative Ct method to calculate changes in Ct, and, ultimately, fold and percent change. An average Ct value for each RNA was obtained for replicate reactions.

### 4.5. miRNA Data Analysis

Hierarchical cluster and heat map analyses were performed using the MultiExperiment Viewer (MeV v4.8) program of TM4 Microarray Software Suite. Heat maps of miRNAs versus pathways were generated using miRPath v3.0 database as previously described [52]. Information concerning MiRNA, mRNA target and related pathways was obtained from the literature and miRBase and Targetscan databases [53].

DIANA-miRPath v3.0 is based on a new relational schema, specifically designed to accommodate this as well as future miRPath updates. miRNA and pathway-related information was obtained from miRBase 18 [54] and Kyoto Encyclopedia of Genes and Genomes (KEGG) v58.1 [55].

Hierarchical clustering of targeted pathways and miRNAs was realized using DIANA-miRPath v3.0. The software created a clustering of the selected miRNAs based on their influence on molecular pathways [52].

### 4.6. Determination of Soluble PD-L1 and PD-1 Concentrations in Plasma

The plasma sPD-1 and sPD-L1 levels have been previously measured using specific homemade ELISA assays not yet commercially available, designed according to the investigator specifications, as previously described [47,56,57].

### 4.7. Statistical Analysis

Filtering criteria able to select reliably quantifiable miRNAs were used (cut off <35 Ct). Undetermined values of Ct were estimated as 40 Ct (the last cycle of the reactions). Heat maps were constructed using z-transformed relative gene Ct values, so that measurements were scaled to obtained gene-wise zero mean and unit variance. Data are represented as mean value ± S.D (standard deviation). Statistical analysis for miRNAs were performed by Student’s t-test. Values of *p* value < 0.05 were considered to be statistically significant.

One-way analysis of variance (ANOVA) was used to perform a study of correlation between plasma immune checkpoint levels in metastatic mccRCC patients before and after nivolumab treatment.

## 5. Conclusions

Our study, for the first time, analyzed the miRNA expression profile in the peripheral lymphocytes of mRCC patients, showing the particular induction of a specific subset of miRNAs in RCC patients with a longer response (>18 months) to nivolumab treatment. We also provide the evidence that miRNAs represent an additional level of regulation of immune checkpoint expression. The synergy of more miRNAs could have a clinical value, supporting the development of combinatorial miRNA signatures as clinical tools. These findings could help to identify novel dynamic predictive biomarkers urgently needed to predict the potential response to immunotherapy and to guide clinical decision-making in RCC patients.

## Figures and Tables

**Table 1:** Clinical and pathological features of metastatic renal cell carcinoma (mRCC) patients.

Baseline Characteristics	
Tot (No.)	23
Median Age (range)—years	62 (36–71)
Sex, *n*. (%)	
Male	19 (82.6%)
Female	4 (17.4%)
Histological classification	
Clear cell	23 (100%)
Other	0 (0%)
Prior nephrectomy	
Yes	7 (30.4%)
No	16 (69.6%)
No. of evaluable disease sites, *n* (%)	
≤2	5 (19.1%)
≥3	18 (80.9%)
Site of metastasis	
Lung only	7 (30.4%)
Lung + others	16 (69.6)
Site of metastasis, individual	
Lung	11 (47.8%)
Lymph node	12 (52.2%)
Liver	6 (26.1%)
Bone	5 (21.7%)
Pancreas	1 (4.3%)
SNC	4 (17.4%)
IMDC Prognostic Risk Group, *n* (%)	
Favorable	6 (26.1%)
Intermediate	17 (73.9%)
Poor	0 (0%)
Best response to nivolumab treatment	
Complete Response (CR)	1 (4.3%)
Partial Response (PR)	9 (39.2%)
Stable Disease (SD)	11 (47.8%)
Progressive Disease (PD)	2 (8.7%)
Median duration of response (range) -months	15 (3−29)

**Table 2:** microRNAs induced by Nivolumab treatment.

microRNAs			
miR-99a	miR-708	miR-655	miR-582-3p
miR-492	miR-487a	miR-485-3p	miR-449a
miR-433	miR-431	miR-429	miR-376c
miR-342-5p	miR-340	miR-339-5p	miR-335
miR-324-5p	miR-25	miR-24	miR-22
miR-221	miR-214	miR-194	miR-18b
miR-152	miR-143	miR-100	miR-let-7e

**Table 3:** Cellular pathways modulated by 28 specific miRNAs induced by Nivolumab.

Pathway	miRNAs	No. of Gene Targets
PI3K-Akt signaling	26	183
MAPK signaling	24	130
T-cell receptor signaling	24	55
Hippo signaling	22	87
FOXO signaling	22	75
HIF-1 signaling	22	56
mTOR signaling	20	35

The list of pathways was obtained using DIANA-miRPath v3.0. The first column reports the pathway statistically relevant, the middle column shows the number of miRNAs involved in the same pathway, and the last column reports the number of the target genes. *p* < 0.005.

**Table 4:** microRNAs induced by nivolumab treatment.

miRNAs	Mean Ct Value	Gene Targets
**miR-22**	12.86	AKT3
**miR-24**	7.80	MAPK1
**miR-99a**	17.63	GSK3B, GIMAP1, TMEM71, TIAM1, PDE4D, TRABD2A, SCGN, CUX1, CD93, EIF1, HIPK1, FNTA, LRRC28
**miR-194**	16.74	STAT6, PCDHA4, IRF1, NOS1
**miR-214**	16.81	PIK3CB, PAK3, PAK6
**miR-335**	17.82	MET, SOS2, PIK3CB, PAK2/3, TGF-A, AKT3, ETS1, PIK3R1, PIK3CG, PIK3CA, CREBBP, EGLN1
**miR-339**	15.77	PIK3CB, SOS1, PAK6, VEGFA
**miR-708**	17.81	SOS2, CRK, CDC42

## Supplementary Materials

The following are available online at https://www.mdpi.com/2072-6694/12/11/3396/s1, Table S1: Hypothetical gene targets of 21 induced-therapy miRNAs involved in clear cell kidney cancer pathways, Figure S1: KEGG pathway related to renal cell carcinoma modulated by the “lymphocyte signature” of 8 miRNAs.
