# Supplementary material for: A “Lymphocyte MicroRNA Signature” as Predictive Biomarker of Immunotherapy Response and Plasma PD-1/PD-L1 Expression Levels in Patients with Metastatic Renal Cell Carcinoma: Pointing towards Epigenetic Reprogramming
Source: Cancers (Basel). 2020 Nov 16;12(11):3396. doi: 10.3390/cancers12113396 (PMC7697734; doi:10.3390/cancers12113396)
Supplement: Supplementary file 1 [file cancers-12-03396-s001.pdf]

cancers

Supplementary Materials

A "Lymphocyte MicroRNA Signature" as Predictive Biomarker of Immunotherapy Response and Plasma PD-1/PD-L1 Expression Levels in Patients with Metastatic Renal Cell Carcinoma: Pointing towards Epigenetic Reprogramming

Lorena Incorvaia, Daniele Fanale, Giuseppe Badalamenti, Chiara Brando, Marco Bono, Ida De Luca, Laura Algeri, Annalisa Bonasera, Lidia Rita Corsini, Salvatore Scurria, Juan Lucio Iovanna, Antonio Russo and Viviana Bazan

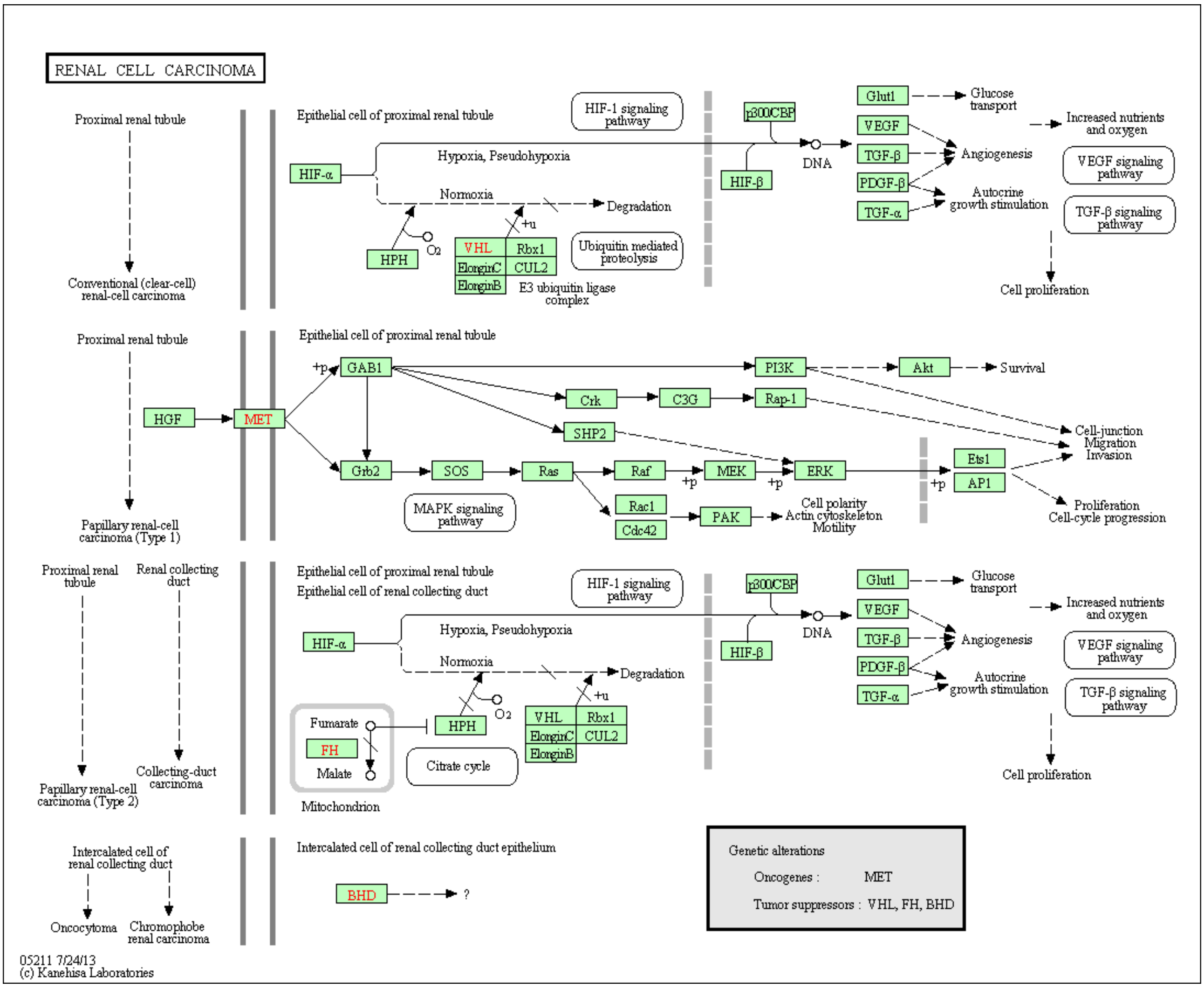

Figure S1. KEGG pathway related to renal cell carcinoma modulated by the "lymphocyte signature" of 8 miRNAs.

Table 1. Hypothetical gene targets of 21 induced-therapy miRNAs involved in clear cell kidney cancer pathways.

**Clear cell renal cancer signaling pathways**			
BRAF	MET	SOS2	RAPGEF1
NRAS	CRKL	CRK	PIK3CB
CUL2	PAK2	TGFA	RAP1A
ARNT	ETS1	PAK7	KRAS
PAK3	VHL	EPAS1	ARAF
JUN	PIK3R3	HIF1A	EGLN3
PIK3R1	SOS1	PIK3CG	PTPN11
RAC1	CDC42	PAK6	TGFB2
EP300	GAB1	AKT3	PAK6
PIK3CA	MAP2K1	SLC2A1	HGF
VEGFA	MAPK1	CREBBP	RAP1B
EGLN1			

The table represents 45 supposed target genes of 21 miRNAs specifically induced by Nivolumab in mRCC patients. The list was generated by DIANA-miRPath v3.0. $p<0.005$
